# Rewriting the byline: toward epistemic justice in global health authorship

**DOI:** 10.3389/fpubh.2025.1621979

**Published:** 2025-10-10

**Authors:** Animesh Ghimire

**Affiliations:** ^1^Sustainable Prosperity Initiative Nepal, Kathmandu, Nepal; ^2^Faculty of Medicine, Nursing and Health Science, Monash University, Clayton, VIC, Australia

**Keywords:** global health, authorship, epistemic justice, LMICs, decolonization, bioethics, equity, publication ethics

## 1 Introduction: a dual perspective on a fractured landscape

How can global health interventions truly serve those most in need if the researchers closest to these challenges are systematically marginalized? This question is central to the development of global bioethics and highlights the critical importance of equitable authorship in global health research. Drawing on my experiences in Nepal—where I witness firsthand the ingenuity of local researchers operating under severe resource constraints—and in Australia, where academic success depends on navigating grants and high-impact publishing, I have come to recognize a disturbing reality: researchers from LMICs are routinely sidelined, their expertise undervalued, and their contributions overshadowed by systemic biases.

This is more than an issue of representation. Following Fricker (1, 2), I use **epistemic injustice** to denote patterned harms to knowers—encompassing *testimonial injustice* (unwarranted credibility deficits) and *hermeneutical injustice* (gaps in shared interpretive resources)—that prevent LMIC scholars from shaping agendas, analytic frames, and the uptake of results. In this article, **global health authorship** refers to authorship and credit practices across public-health, clinical, and health-policy journals addressing LMIC populations. I refer to the concept of an **epistemic extraction economy** to describe a recurring process in which knowledge produced in LMICs is sourced, recontextualized, and valorized within high-income country (HIC) institutions. In this dynamic, decision-making power and rewards tend to flow predominantly upstream, creating an analytical distinction from collaboration. In this context, control over conceptual framing and the distribution of benefits remains asymmetrical. By shaping research priorities, methodologies, and interpretations without equitable input from LMIC scholars, current authorship practices violate core bioethical principles of justice, equity, and respect for persons. Building on Elzinga's account of **epistemic drift** (3, 4), I later introduce ***Generational Epistemic Drift*** to capture cohort-by-cohort shifts within LMIC institutions that normalize external paradigms and displace locally anchored methods. This paper contends that the current approach necessitates not only incremental reforms but also a comprehensive reassessment of the processes through which global health knowledge is generated, validated, and disseminated.

## 2 The illusion of meritocracy: power dynamics in publication

In theory, academic success in global health research rests on a meritocratic foundation: the most rigorous studies secure prestigious grants, earn prime authorship slots in high-impact journals, and ultimately shape policy (5). Yet this narrative masks a far more insidious reality. From the moment a research question is conceived, power imbalances inherited from colonial histories and perpetuated by contemporary funding and publishing structures influence whose voices are heard, whose data are deemed valuable, and whose perspectives shape the global health agenda.

### 2.1 Reinforcing colonial legacies through metrics and networks

Merit in academic publishing is often measured by quantifiable outputs—impact factors, citation counts, and grant totals—all metrics that disproportionately favor HIC institutions. Top-tier journals are frequently headquartered in the Global North, with editorial boards and peer reviewers with limited exposure to LMIC contexts (6). This “clustering effect” nurtures self-reinforcing networks, wherein prior associations and institutional prestige weigh more heavily than the intrinsic value of local knowledge (7). Promotion frameworks at leading United States (US) universities explicitly weight first- and senior/last-authored papers in high-impact journals, citation indices, Principal Investigator (PI) leadership, and sustained extramural funding as markers of “national recognition,” thereby codifying the same merit signals that cluster in HIC networks (8). Furthermore, evidence from economics indicates that authors in developing countries publish in top-tier journals less frequently, even when comparing papers with similar citation counts. Additionally, their work garners fewer citations overall (9). LMIC scholars without access to these elite networks, or to costly conferences where such networks are forged, begin at a systemic disadvantage long before submission.

### 2.2 Barriers woven into the fabric of research production

Institutional constraints intensify these network effects. LMIC researchers often lack protected research time, adequate administrative support, and equitable research budgets. Chronic underfunding of infrastructure—from laboratories to reliable internet—further limits their ability to produce the kinds of outputs prioritized by HIC-dominated editorial circles (10). These constraints are anchored upstream in funding flows. In 2020, the WHO Global Observatory on Health Research and Development (R&D) reported that low-income countries received only 0.2% of all health-research grant funding, and only 0.2% of non-communicable-disease grants were allocated to institutions in LMICs (11). Major donors concentrate awards within the Global North—~70% of Fogarty grants to US and HIC institutions, 73% of Wellcome Trust grants supporting United Kingdom (UK) based activity, 80% of United States Agency for International Development (USAID) contracts to US firms, and an estimated 88% of Bill and Melinda Gates Foundation (BMGF) grants held by Northern institutions (12). In vaccine R&D specifically, organizations in Asia, Africa, and Central/South America received < 20% of global bacterial-vaccine funding; 16.78% of total funds accrued to LMIC recipients, and when Indian domestic funding is excluded, LMIC receipts fall to 5.91% (13). These patterns are mirrored in human-resource capacity: HICs have about 56 times more health researchers per million inhabitants than LMICs (11). When manuscripts do emerge, linguistic and stylistic norms anchored in Western academic traditions function as gatekeeping devices (14), penalizing work that departs from narrow conventions and imposing additional (often unfunded) language-editing costs.

### 2.3 The hidden curriculum of authorship

Even when LMIC scholars collaborate with HIC partners, authorship negotiations can be subtly skewed. Early-career researchers in LMICs often adopt subordinate roles—data collection, translation, or field coordination—while conceptual leadership and final authorship positions remain concentrated in wealthier institutions (15). In clinical disciplines, continuing medical education (CME) policies further reinforce this hierarchy: the American Medical Association (AMA) grants the Physician's Recognition Award (PRA) for published articles only when the applicant is listed as the first or last author in a PubMed-indexed, peer-reviewed journal (16). This crediting rule systematically rewards lead/senior positions and incentivizes HIC-affiliated researchers to retain—or claim—primary authorship in mixed LMIC–HIC teams. Promotion and tenure criteria at leading U.S. medical schools similarly prioritize first-/senior authorship in high-impact journals, citation impact, and PI-led funding, while middle authorship is discounted unless a “pivotal role” is documented—further channeling career credit toward HIC-based teams (8). Such dynamics reflect an unspoken “hidden curriculum” in global health publishing: to succeed, LMIC scholars must align themselves with the priorities of HIC counterparts, who control the funding and dissemination channels (12). This tacit requirement stifles local innovation, as studies are designed and reported to appease external gatekeepers rather than authentically reflect community-driven research imperatives.

### 2.4 From statistical footnote to epistemic exclusion

These disadvantages are not benign oversights; they translate into measurable patterns of exclusion. In a systematic analysis of global surgery, the majority of authors were affiliated only with HIC institutions (51%), with LMIC-affiliated teams under-represented across seniority strata (17). The pattern persists—even intensifies—within scholarship explicitly concerned with the ethics and practice of decolonization: among 197 publications on “decolonizing global health” and global health partnerships, 70.0% had HIC only bylines, 22.3% were mixed HIC–LMIC, and only 7.6% were LMIC only (18). Together with cross-field evidence from economics on citation penalties and under-representation for LMIC authors (9), these findings show how structural asymmetries in networks and funding culminate in authorship hierarchies that privilege external interpretations over locally grounded analysis (19)—converting LMIC expertise from leadership into a footnote.

## 3 Manifestations of exclusion: beyond tangible resource gaps

Despite widespread recognition of funding shortfalls, linguistic barriers, and high publication fees, these well-known obstacles represent merely the tip of an iceberg (20). Deeper, subtler dynamics—ranging from conceptual appropriation to algorithmic discrimination—collectively produce what might be termed an “***epistemic extraction*** ***economy***,” in which local knowledge from LMICs is mined, repackaged, and valorized within HIC institutions (21). This extraction does not simply reinforce existing inequities in authorship; it actively reshapes the global health research agenda in ways that can undermine local priorities and perpetuate colonial hierarchies of knowledge production.

### 3.1 Conceptual appropriation and erasure

A stark illustration of these subtle power imbalances lies in the appropriation of locally developed theoretical frameworks. While Western research paradigms are esteemed as the “gold standard,” indigenous or context-specific models—such as community-based participatory research methods anchored in cultural practices—are often either dismissed or integrated into projects without giving due credit to their originators (22). Consequently, LMIC scholars see their innovations published through Western lenses, with key insights relabeled or subsumed under universalizing theories (23). This process effectively erases the intellectual lineage of non-Western scholarship, relegating LMIC researchers to the role of data collectors rather than intellectual contributors.

### 3.2 The hidden politics of peer review

Peer review—ostensibly a neutral mechanism for maintaining scholarly rigor—can become yet another site of exclusion when reviewers are ill-equipped to appreciate localized methodologies or cultural nuances (24). Editors and reviewers unfamiliar with region-specific practices may conflate “novelty” or “relevance” with conformity to Western research norms, inadvertently penalizing studies that address community-defined needs rather than internationally trendsetting topics (25, 26). In the worst cases, LMIC-led research is critiqued or downgraded for failing to align with external expectations, even though its methodological choices may be more ethically and pragmatically attuned to the local context (27). Policy bodies—most notably the WHO Global Observatory on Health R&D—have called for funding and evaluation practices aligned with local public-health needs and for systematic tracking of R&D indicators to expose these gaps (11).

### 3.3 Algorithmic gatekeeping in the digital age

As academic publishing becomes increasingly digitalized, LMIC researchers confront the rise of algorithmic sorting systems—used by journals and funding agencies to pre-screen submissions based on citation potential, institutional rankings, or even textual features (28). Early-career scholars from lesser-known institutions may be algorithmically filtered out before human reviewers even see their work, creating a hidden layer of gatekeeping that amplifies existing hierarchies (29). These algorithms, trained primarily on datasets from HIC-dominated research, often fail to recognize indicators of quality in LMIC-led scholarship (30). Unlike the visible barriers of article processing charges (20), algorithmic gatekeeping is intangible yet profoundly consequential, narrowing the pipeline of diverse manuscripts.

### 3.4 Social and emotional costs of “global health tourism”

Beyond financial or technical barriers, a neglected form of exclusion occurs through short-term “global health tourism,” in which researchers from the Global North conduct rapid field studies in LMICs for high-impact publications but fail to build sustained, reciprocal partnerships (31). While communities may initially benefit from material inputs—such as data-collection stipends—these projects frequently end once the researchers depart, leaving local scholars under-resourced, under-credited, and overwhelmed by the unfulfilled promise of future collaboration. Many universities promote these short-term “opportunities” as recruitment incentives, using them to attract fee-paying students and enhance faculty recruitment by offering overseas placements, touting “impact” narratives, and guaranteeing rapid publication trajectories. As a result, LMIC sites become conduits for generating tuition revenue, improving visibility in rankings, and appealing to donors. This practice introduces an additional layer of extraction: local partners provide valuable experiential and reputational capital that predominantly benefits the home institutions, even when the collaborations are not sustained (32). Such extractive practices not only disrupt local research ecosystems but also impose emotional and reputational costs on LMIC investigators, who are left grappling with inflated expectations that often never materialize into lasting scholarly opportunities.

### 3.5 Epistemic extraction as a systemic imperative

**Figure 1:**
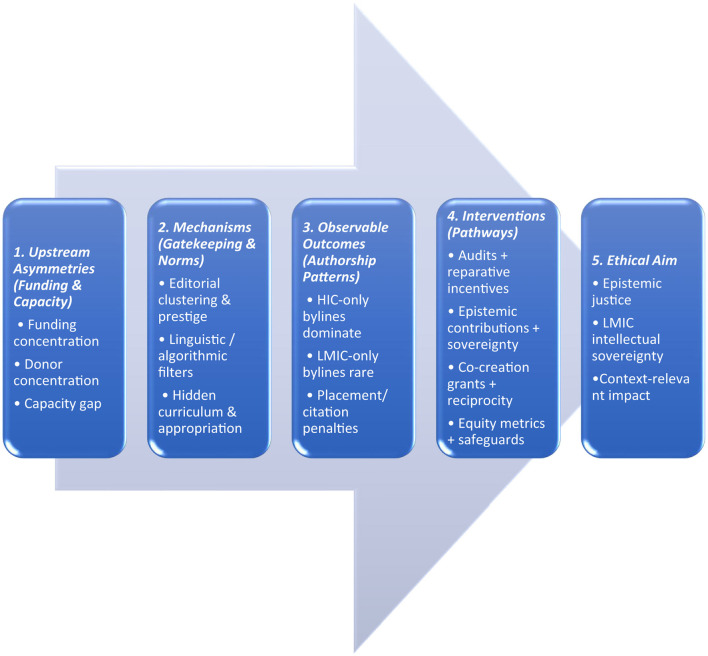
Overview—from upstream asymmetries through mechanisms and outcomes to interventions and the ethical aim of epistemic justice.

When these dynamics come together, they create an ecosystem in which expertise from LMICs is viewed as a valuable source of “authentic” data. However, this expertise is often devalued in important areas such as authorship, editorial oversight, and conceptual framing. Recognizing these financial and status-driven incentives helps explain why this extraction continues, even when scholarly credit is not fairly distributed. This imbalance is not a coincidence; it reflects historical power relations that allow HIC institutions to dominate the definition of what constitutes legitimate knowledge in global health. By portraying resource constraints as the primary challenge, the discourse often obscures the deeper, more intangible forces that sustain and reinforce these material inequities (Figure 1).

## 4 Generational epistemic drift: the erosion of LMIC intellectual sovereignty

Building on Aant Elzinga's notion of epistemic drift (3, 4)—where the criteria for valuing research are increasingly shaped by external, often political or commercial imperatives—this discussion foregrounds a generational dimension that illuminates how such shifts erode the intellectual sovereignty of LMICs. Over successive cohorts of LMIC researchers, locally embedded knowledge frameworks give way to Western theoretical models, not necessarily through overt coercion, but via gradual alignment with norms that promise greater publication success or funding potential. In effect, Generational Epistemic Drift describes the cumulative displacement of region-specific insights as they are recast in Euro-American paradigms, thereby undervaluing and eventually marginalizing ways of knowing that deviate from dominant academic standards. If left unchecked, this process impedes the development of contextually grounded health interventions and reinforces inequities by compelling LMIC scholars to sacrifice authenticity for externally validated scholarly achievements.

## 5 Toward epistemic justice: pathways for action

Confronting the entrenched structures that perpetuate epistemic injustice requires a bold reimagination of how global health research is initiated, funded, evaluated, and disseminated. The following strategies propose incremental reforms and a transformative shift, recalibrating the very architecture of knowledge production:

- **Authorship equity audits with reparative incentives**
  Major global health funders—both in HICs and LMICs—should implement mandatory, publicly transparent ***Authorship Equity Audits*** for all funded projects. Extending beyond a simple tally of authorship positions, these audits would include qualitative assessment of LMIC leadership in design, methods, and interpretation. Where audits reveal significant imbalances, funders should allocate ***reparative incentives***: resources earmarked for local capacity-building and intellectual-leadership training. This dual mechanism identifies root causes and systematically channels resources to LMIC teams, rectifying historical disparities. These audits align with the WHO Observatory's call for routine, systematic R&D data collection to guide equitable allocation (11).
- **Structured epistemic contribution statements with sovereignty clauses**
  In addition to detailed author-contribution lists, journals should adopt ***Structured*** ***Epistemic Contribution Statements*** attributing specific forms of intellectual labor—local contextual insights, culturally situated methods, or indigenous ethical frameworks—to LMIC co-authors. To address appropriation risk, these statements should incorporate ***Sovereignty Clauses***, legally binding provisions that protect LMIC-driven innovations from being repackaged without appropriate credit or control. This approach moves beyond transparency to establish enforceable safeguards against epistemic exploitation.
- **Knowledge co-creation partnership grants with reciprocity mandates**
  Funders should launch ***Knowledge Co-Creation Partnership Grants*** that tie eligibility to evidence of equitable collaboration from the proposal stage. Beyond shared budgets and joint data ownership, applicants must specify ***reciprocity mandates***—long-term, two-way exchanges of expertise—and credible local dissemination plans (open-access local-language outputs, stakeholder roundtables, and community-led policy briefs). Reciprocity requirements help redirect currently externalized funding flows toward LMIC leadership and local dissemination (11, 13).
- **Epistemic equity metrics in institutional assessment with generational safeguards**
  Universities and research institutes—especially in HICs—should adopt equity metrics in performance reviews, promotion criteria, and partnership agreements, and pair them with ***Generational Safeguards*** that keep locally anchored, community-driven methods in active use across cohorts. Metrics should show: (i) leadership by scholars from LMICs (e.g., share of LMIC first/corresponding authors and co-principal investigators), (ii) co-creation (documented shared design and decision rights), and (iii) local-language and community dissemination. These measures complement funder-level tracking recommended by the WHO Observatory (11) and help ensure that LMIC ways of knowing are retained, rewarded, and reproduced rather than gradually replaced.

## 6 Implementation backbone: feasibility, resistance, and precedents

The proposed measures outlined in the previous section are operationally feasible, as they can be integrated into existing infrastructure utilized by funders, journals, and universities. Grant-reporting portals currently capture Principal Investigator (PI) metadata and budget allocations; journal submission systems have standardized contributor-role taxonomies (such as CRediT) and data availability statements; and promotion dossiers already document outputs and partnerships. A streamlined framework that incorporates standardized fields, annual aggregated reporting, de-identified public dashboards, and random sample verification effectively reduces the associated burden while ensuring that equity remains open to audit. Potential sources of resistance may include administrative challenges, which can be alleviated through the use of templated forms and machine-readable exports; legal concerns related to credit and control, addressed by model contract addenda that have been thoroughly vetted for reuse; reputational risks, managed through the implementation of corrective plans instead of sanctions; and the possibility of metric manipulation, which can be mitigated through independent checks and triangulation. It is important to note that similar systems have already successfully driven behavior change at scale: accreditation cycles for Institutional Review Boards/Research Ethics Committees (IRB/REC) have normalized ethics oversight; journal mandates for trial registration have established norms for prospective registration; contributor-role taxonomies have rapidly been adopted by publishers; and Product Development Partnerships (PDPs) illustrate how cross-institutional governance can direct resources toward the priorities of low- and middle-income countries. These precedents suggest that the pathways to actionable measures are not only feasible and verifiable, but also capable of generating near-term change.

## 7 Conclusion

The politics of authorship in global health is not a matter of procedural protocol but a litmus test for the field's commitment to equity and respect for persons. By privileging certain epistemologies and undervaluing local expertise, current practices undermine the promise of universal health improvements and perpetuate colonial legacies in new guises. The structural shifts outlined above—ranging from reparative incentives to enforceable sovereignty clauses—urge us to take the notion of co-liberation seriously, where LMIC researchers are included and empowered as equals in setting the agenda and interpreting the data that shape policy and practice.

Ultimately, achieving epistemic justice in global health demands more than good intentions; it requires systemic transformation that holds funders, journals, institutions, and researchers accountable for dismantling entrenched hierarchies. Equipping LMIC scholars with intellectual and structural autonomy is pivotal to fostering innovative, context-driven solutions that respond more effectively to the profound health challenges facing diverse communities worldwide. As we collectively undertake these shifts, we must reckon with a deeply consequential question: How can global health scholarship be re-envisioned so that the knowledge of LMIC researchers is not only recognized but also shapes the very foundations of what we consider valid, rigorous, and transformative science?
